# Highly sensitive detection of the *PIK3CA*^*H1047R*^ mutation in colorectal cancer using a novel PCR-RFLP method

**DOI:** 10.1186/s12885-016-2493-9

**Published:** 2016-07-12

**Authors:** Wan-Ming Li, Ting-Ting Hu, Lin-Lin Zhou, Yi-Ming Feng, Yun-Yi Wang, Jin Fang

**Affiliations:** Department of Cell Biology, Key Laboratory of Cell Biology, Ministry of Public Health, and Key Laboratory of Medical Cell Biology, Ministry of Education, China Medical University, No.77 Puhe Road, Shenyang North New Area, Shenyang, Liaoning Province 110122 People’s Republic of China

**Keywords:** *PIK3CA*, Low abundance mutation, Colorectal cancer, PCR-RFLP, Targeted therapy

## Abstract

**Background:**

The *PIK3CA*^*H1047R*^ mutation is considered to be a potential predictive biomarker for EGFR-targeted therapies. In this study, we developed a novel PCR-PFLP approach to detect the *PIK3CA*^*H1047R*^ mutation in high effectiveness.

**Methods:**

A 126-bp fragment of *PIK3CA* exon-20 was amplified by PCR, digested with FspI restriction endonuclease and separated by 3 % agarose gel electrophoresis for the PCR-RFLP analysis. The mutant sequence of the *PIK3CA*^*H1047R*^ was spiked into the corresponding wild-type sequence in decreasing ratios for sensitivity analysis. Eight-six cases of formalin-fixed paraffin-embedded colorectal cancer (CRC) specimens were subjected to PCR-RFLP to evaluate the applicability of the method.

**Results:**

The PCR-RFLP method had a capability to detect as litter as 0.4 % of mutation, and revealed 16.3 % of the *PIK3CA*^*H1047R*^ mutation in 86 CRC tissues, which was significantly higher than that discovered by DNA sequencing (9.3 %). A positive association between the *PIK3CA*^*H1047R*^ mutation and the patients’ age was first found, except for the negative relationship with the degree of tumor differentiation. In addition, the highly sensitive detection of a combinatorial mutation of *PIK3CA*, *KRAS* and *BRAF* was achieved using individual PCR-RFLP methods.

**Conclusions:**

We developed a sensitive, simple and rapid approach to detect the low-abundance *PIK3CA*^*H1047R*^ mutation in real CRC specimens, providing an effective tool for guiding cancer targeted therapy.

## Background

The phosphatidylinositol 3-kinases (PI3Ks) are a large family of lipid kinases, and play an important role in many cellular processes, such as cell survival, proliferation, and migration [1, 2]. *PIK3CA*, encoding for the catalytic subunit p110-alpha of class I PI3Ks, is a member of this lipid kinase family. It is reported that mutant *PIK3CA* contributes to tumorigenesis through increased tumor invasion, decreased apoptosis and loss of contact inhibition [3, 4]. More than 30 % of various human cancer types were found to contain mutations in the *PIK3CA* gene, and it is frequently mutated in cancers of the liver, breast, stomach, breast, lung, and colon [5, 6].

Recently, several studies have revealed that *PIK3CA* mutations are associated with a negative prediction for targeted therapy by anti-EGFR MoAb (panitumumab or cetuximab) [7, 8]. In the case of colorectal cancers (CRC), apart from *KRAS* and *BRAF*, which have been proven to be significant predictive markers of the anti-EGFR MoAb response [9], the *PIK3CA* exon-20 (H1047R) point mutation is likely to a potential predictive biomarker of personalized therapy for CRC [10, 11]. De Roock et al. showed that the *PIK3CA*^*H1047R*^ mutation was associated with a worse outcome compared with wild-type, with a targeted therapy response rate of 0.0 % versus 36.8 %, respectively [8]. Therefore, the effective detection of the *PIK3CA*^*H1047R*^ mutation is increasingly important to accurately predict and guide individualized therapy.

To date, DNA sequencing is considered to be the gold standard for gene mutation screening, but it is mainly limited by low sensitivity (20–30 %) for the clinically low abundance mutations, resulting in incorrect groupings and improper clinical therapy [12]. Although the rapidly developed next-generation sequencing technology provides increased detection sensitivity (5 %) [13], the advantages of this technology must be further elicited before it is routinely used. Other methods, such as HRM, have a higher sensitivity and less sample contamination, but the requirement for special equipment and an additional sequencing confirmation step limit their universal application in clinical settings [14, 15]. Digital PCR has the potential to offer more sensitive and considerably more reproducible clinical methods, but is as susceptible to upstream errors associated with factors such as sampling and extraction, and also suffers systematic bias [16]. Thus, there is an urgent need to develop a method that possesses higher detection efficiency and is suited to routine usage in the laboratory to screen for low-abundance mutations.

Polymerase chain reaction-restriction fragment length polymorphism (PCR-RFLP) analysis is a widely applied method to detect gene mutations, which allows distinguishing mutant-type and wild-type sequences via destructing or generating enzyme restriction sites through PCR and subsequent electrophoresis separation of differential fragments [17]. Compared to other methods, PCR-RFLP offers a simple operation, higher sensitivity and reproducibility, and no complex equipment requirements [18, 19]. For *KRAS* exon-2 mutations, the sensitivity of the PCR-RFLP method was at least 0.1 % [20]. More importantly, it is preferentially suitable to detect point mutations [21].

For CRC, RFLP methods have been used for the detection of targeted therapy-related *KRAS* and *BRAF* gene mutations, and the corresponding *KRAS* mutation assay kit is commercially available [20, 22]; however, no PCR-RFLP method has been developed for *PIK3CA*^*H1047R*^. Several clinical trials and retrospective studies have suggested that the combinatorial detection of *KRAS* and *BRAF* mutations could increase positive mutation detection and therefore improve therapy response rates [23]. However, recent research showed that some patients carrying wild-type *KRAS* and *BRAF* still do not respond to anti-EGFR MoAbs, among which *PIK3CA*^*H1047R*^ mutation carriers were found [24, 25]. Therefore, the combinatorial detection of these three gene mutations might increase the response rates. Tian et al. analyzed *KRAS*, *BRAF* and *PIK3CA* mutations in 381 CRC samples in combination, achieving improved treatment classification and increased response rates [26]. In addition, the current evidence about relationship of *PIK3CA* mutation and the targeted therapeutic effect is mostly dependent on the relatively low sensitivity methods, such as direct sequencing, which may result in inaccurate information [27, 28]. Accordingly, in this study, we developed a specific, fast and simple PCR-RFLP method for detecting low-abundance *PIK3CA*^*H1047R*^ mutations by creating an FspI restriction endonuclease recognition site to distinguish wild- and mutant-type *PIK3CA*^*H1047R*^. In sensitivity studies, the PCR-RFLP method presented the capability to detect as little as 0.4 % of the mutant-type fragment in the presence of the wild-type fragment. In 86 paraffin-embedded CRC tissues, the method could detect at least 1.5 % of the *PIK3CA*^*H1047R*^ mutation, which was far below that of direct sequencing, and statistical analysis revealed that the *PIK3CA*^*H1047R*^ mutation was associated with patients’ age and tumor differentiation. To explore the possibility of detecting multi-gene mutations in combination using PCR-RFLP, the mutations of three target-EGFR genes, including *KRAS*, *BRAF* and *PIK3CA* in CRC tissues were detected using individual PCR-RFLP methods.

## Methods

### Cell lines

The human colorectal cancer cell lines LoVo, SW620, LS174T, HT29, HCT-8 and Colo205 were maintained in RPMI1640 containing 10 % fetal bovine serum (FBS, Invitrogen, Carlsbad, CA, USA) and 100 units/ml penicillin-streptomycin (Sigma-Aldrich, St Louis, MO, USA), and the human colorectal cancer cell lines RKO, CL187, CX-1 and CloneA were maintained in high-glucose DMEM containing 100 units/mL penicillin-streptomycin and 10 % FBS. All of the cells were cultured at 37 °C under a 5 % CO_2_ atmosphere. LoVo and SW620 cells were known to be wild-type for *PIK3CA*^*H1047R*^, while LS174T and RKO cells possessed heterozygous mutations [29].

### Clinical samples

A total of 86 formalin-fixed paraffin-embedded (FFPE) tissue sections (5 μm) from CRC patients were supplied by China Medical University (Shenyang, China). The study was approved by the ethics committee of China Medical University and all of the patients who provided tumor samples provided written informed consent. The patients’ characteristics were collected from the 86 CRC patients, including age, tumor differentiation, gender, tumor size, tumor location, Dukes stage and lymph node status.

### Genomic DNA extraction and PCR-RFLP analysis

Genomic DNA (gDNA) was extracted from the human colorectal cancer cell lines (1 × 10^6^ cells) using the Genomic DNA Purification Kit (Promega, US) according to the manufacturer’s instructions. All DNA templates were eluted with 40 μl ddH_2_O and stored at −20 °C until use. The purity and concentration of extracted DNA were determined by spectrophotometry (NanoDrop 2000, Thermo Fisher Scientific Inc., USA). The DNA samples with absorption ratios of 260/280 nm greater than 1.8 were for subsequent analyses. Subsequently, PCR amplification was performed using 50 ng of gDNA as template for the analysis of *PIK3CA*^*H1047R*^ mutation statuses.

**Fig. 1:**
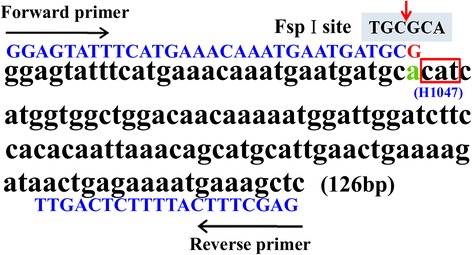
The nucleotide sequence design for the detection of the *PIK3CA* ^*H1047R*^ mutation by PCR-RFLP. A 126-bp fragment covering *PIK3CA* exon-20 was chosen from the human genome for PCR amplification. Primer sequences are highlighted in blue. The forward primer sequence harbors one mismatched site (a → G, shown in red) to creating a new TGCGCA sequence for the FspI restriction endonuclease recognition site

The 126-bp fragment of the *PIK3CA* gene covering exon-20 sequences containing the H1047R mutation site is shown in Fig. 1. The PCR designed primers used were: forward, 5′-GGAGTATTTCATGAAACAAATGAATGATGCG-3′ (mismatched nucleotide is underlined), and reverse, 5′-GAGCTTTCATTTTCTCAGTTATCTT-3′. The mismatch forward primer harbored one mismatched site (a → G, Fig. 1, shown in red) to introduce a new TGCGCA sequence for the FspI restriction endonuclease recognition site. In the wild-type *PIK3CA* exon-20, the 126-bp fragment could be digested into 96- and 30-bp fragments. In contrast, *PIK3CA*^*H1047R*^ mutant alleles were not cleaved due to the substitution of CAT to CGT, resulting in the loss of the FspI-recognized site. The PCR reaction was performed using the following cycling conditions: 94 °C for 45 s, 60 °C for 45 s, and 72 °C for 45 s, 30 cycles. The predicted PCR product size was 126 bp and confirmed by electrophoresis in 3 % agarose gel containing ethidum bromide. Typically, 2 μl of 126-bp PCR products of *PIK3CA* were digested with 1 unit of restriction endonuclease FspI in 10 μl at 37 °C for 10 min. The DNA fragments were analyzed by 3 % agarose gel electrophoresis.

### Detection specificity of the PCR-RFLP method

In addition to the H1047R (CAT → CGT) mutation, *PIK3CA* exon-20 may clinically harbor the H1047L mutation (CAT → CTT) [8]. To examine whether the *PIK3CA*^*H1047L*^ mutation could also be resolved by our method, we synthesized the 126-bp sequence containing the H1047L mutant sequence. In addition, the sequences containing the wild-type exon-20 (CAT) and *PIK3CA*^*H1047R*^ mutant (CGT) were also synthesized. These synthesized DNAs were amplified by PCR, digested with FspI, and then electrophoresed on a 3 % agarose gel.

### Detection sensitivity of the PCR-RFLP method

To perform the sensitivity analysis, we obtained wild-type and homozygous mutant-type model sequences by separating heterozygous mutation-type *PIK3CA*^*H1047R*^ derived from the LS174T cells’ genome using TA cloning. Firstly, the LS174T cells’ 126-bp PCR product was cloned into the TA vector using the TA cloning kit (Takara, Japan). Ten bacterial clones were selected and their plasmids were extracted using the QIAGEN Plasmid Mini kit (QIAGEN, Germany) according to the manufacturer’s instructions. After their inserts were sequenced, the homozygous mutant plasmid containing the *PIK3CA* gene was mixed with the wild-type plasmid at the decreasing ratios of 1:1, 1:2, 1:4, 1:16, 1:32, 1:64, 1:128, 1:256, and 1:512, respectively. Subsequently, the mixed plasmid was subjected to PCR-RFLP analysis.

### GDNA extraction from FFPE tissue and mutation detection

FFPE tumor blocks were cut into 5-μm sections and the sections with tumor area more than 70 % were dissected for the study. For gDNA extraction, one 5-μm thick section was used for each case. GDNA was extracted from FFPE tissue samples using the FFPE DNA Kit (OMEGA, USA) according to the manufacturer’s instructions. All DNA templates were eluted with 20 μl ddH_2_O and stored at −20 °C until use.

In order to investigate the applicability of our PCR-RFLP method, we first detected the *PIK3CA* gene mutation status in 86 FFPE CRC tissue sections. The wild-type *PIK3CA* exon-20 of the section was cleaved into two fragments of 96- and 30-bp, while the mutant type remained intact (126 bp). To explore the possibility of detecting CRC targeted therapy-related genes in combination, six samples were chosen to further detect the *KRAS* and *BRAF* mutant status by the PCR-RFLP methods. The primers were synthesized by Sangon Biotechnology Co. Ltd. (Shanghai, China) according to previous reports [30, 31]:

- For *KRAS* (107 bp)
- Forward: 5′-GACTGAATATAAACTTGTGGTAGTTGGACCT-3′.
- Reverse: 5′-CTATTGTTGGATCATATTCGTCC-3′.

After amplification, the fragment of 107 bp was digested by MvaI. The wild-type *KRAS* exon-2 allele were cleaved into two fragments of 77- and 30-bp, while the mutant type remained intact (107 bp).

- For *BRAF* (224 bp)
- Forward: 5′-TCATAATGCTTGCTGATAGGA-3′.
- Reverse: 5′-GGCCAAAAATTTAATCAGTGGA-3′.

After amplification, the fragment of 224 bp was digested by TspI. The wild-type *BRAF* exon-15 allele were cleaved into three fragments of 124-, 87- and 13-bp, while the mutant type yielded only two fragments of 211- and 13-bp.

### Sequencing

To confirm the PCR-RFLP results, sequencing analysis was performed in all samples. All PCR products of the *PIK3CA, KRAS* and *BRAF* genes were directly sequenced to confirm the mutation status using ABI 3730xl DNA Analyzer (Sangon Biotechnology Co. Ltd., Shanghai, China).

For the samples that showed a mutation band in agarose gel electrophoresis but were not detectable by direct sequencing, clone sequencing was performed by the TA cloning kit.

### Statistical analysis

Statistical analysis was carried out using IBM SPSS 20.0 (IBM Corporation, Armonk, NY, USA). Significant differences between groups were assessed using the *χ*^2^ test considering the *P* value as obtained by Fisher’s exact test. A *P* value of less than 0.05 was considered statistically significant differences.

## Results

### Establishment of PCR-RFLP method for the detection of *PIK3CA*^*H1047R*^

**Fig. 2:**
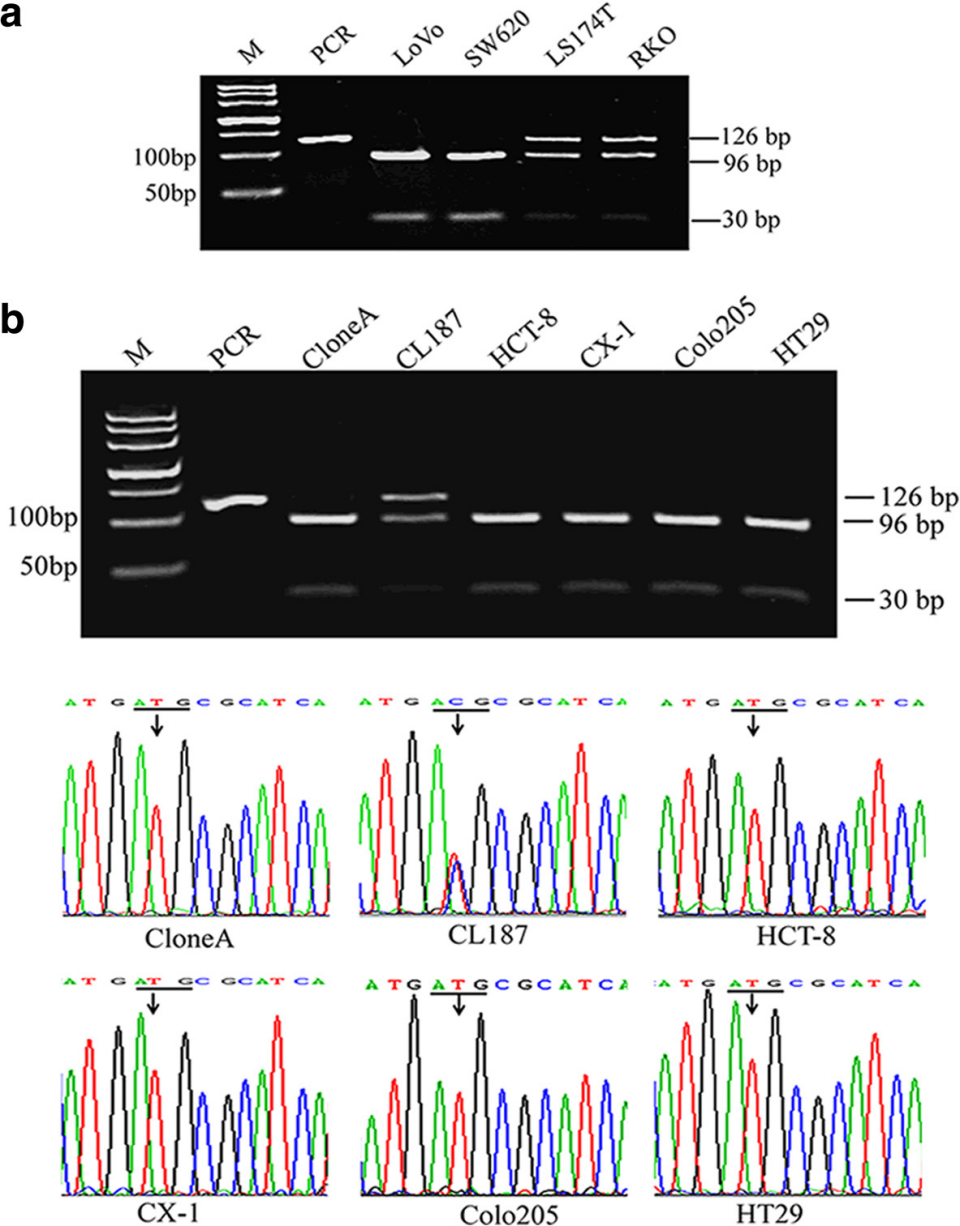
Detection of the *PIK3CA* ^*H1047R*^ mutation in CRC cell lines using PCR-RFLP. **a**: Detection of four CRC cell lines with known *PIK3CA* gene status using PCR-RFLP. The fragment of 126-bp was amplified from the cells’ gDNA, digested with FspI, and then electrophoresed in a 3 % agarose gel. The FspI digestion of wild-type *PIK3CA* yields two bands of 96- and 30-bp, while the mutant-type remains intact (126 bp). **b**: The detection of six CRC cell lines with unknown *PIK3CA* gene status by PCR-RFLP (top) and direct sequencing (bottom). M: DL500 DNA marker

We chose the *PIK3CA* gene 126-bp fragment containing the H1047R mutation site and introduced a new TGCGCA sequence for an FspI restriction endonuclease recognition site by designing a specific mismatch primer to substitute a with G (Fig. 1). The mutant and wild-type sequences are distinguishable based on the difference in size and number of the endonuclease-digested fragment, as the wild-type fragments were 96 and 30 bp, while the mutant fragment was 126 bp. First, we used the PCR-RFLP method to detect CRC cell lines with a known *PIK3CA* gene status. As shown in Fig. 2a, LoVo and SW620 presented in two enzyme-digested fragments with sizes of 96 and 30 bp, revealing the wild-type *PIK3CA*; while LS174T and RKO showed two fragments 96- and 30-bp coexisting with a126-bp fragment, indicating the heterozygous *PIK3CA*^*H1047R*^. All of these results are consistent with previous reports [30].

To further study the applicability of the PCR-RFLP method, we used this method to detect six CRC cell lines whose *PIK3CA* gene status was not reported. Fig. 2b (top) shows that CloneA, HCT-8, CX-1, Colo205 and HT29 cells were cleaved into two fragments with sizes of 96 and 30 bp, which indicated that there were no *PIK3CA*^*H1047R*^ mutations. For CL187 cells, a 126-bp fragment besides 96- and 30-bp fragments was detected, indicating the heterozygous-type *PIK3CA*^*H1047R*^. The results from PCR-RFLP used to detect the *PIK3CA* status in six CRC cell lines were completely consistent with those obtained by direct sequencing (Fig. 2b, bottom).

### Specificity and sensitivity of the PCR-RFLP method

**Fig. 3:**
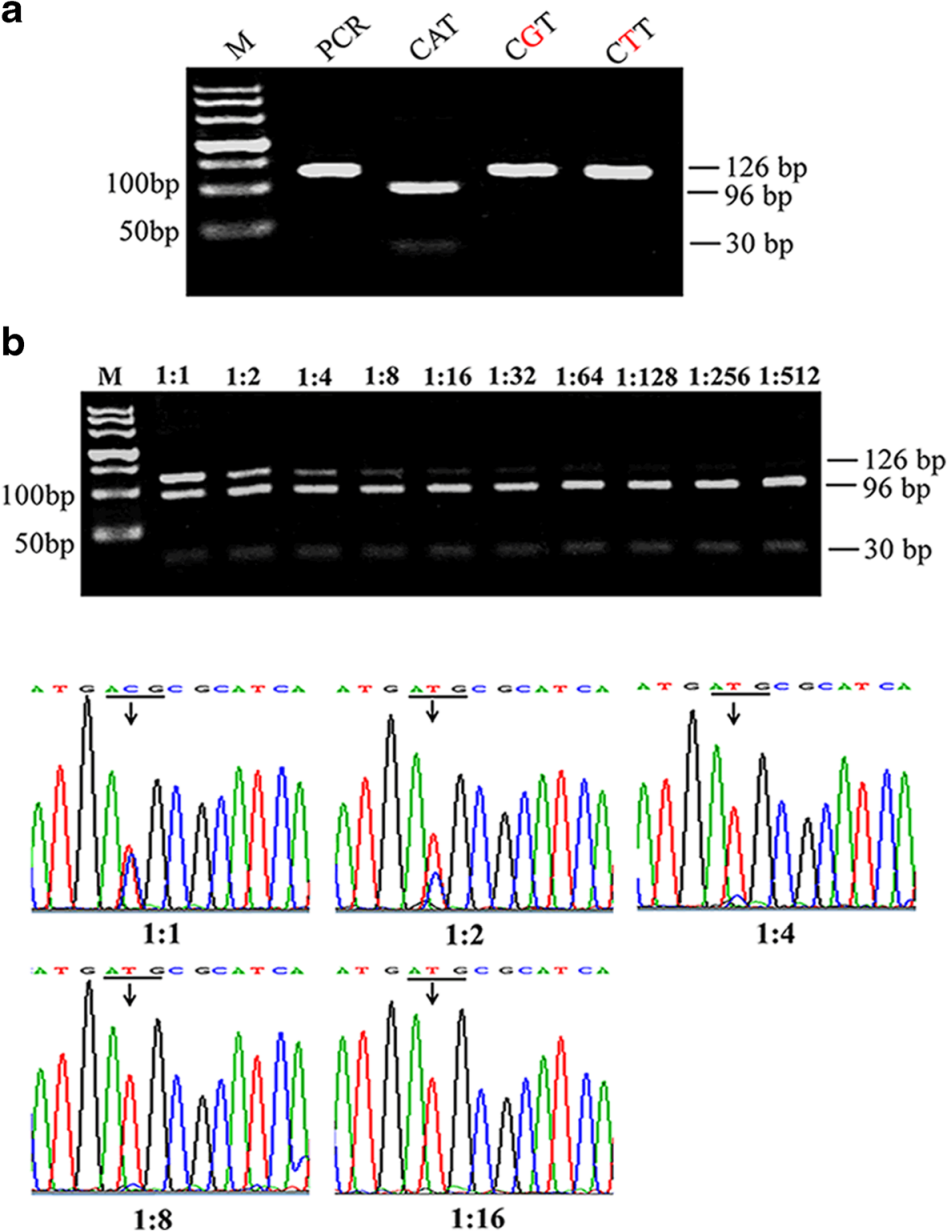
**a**: Detection specificity of PCR-RFLP. Synthetic oligonucleotide sequences containing wild-type *PIK3CA* (CAT) and mutant-type *PIK3CA* (CGT or CTT) were subjected to PCR-RFLP. **b**: Detection sensitivity of PCR-RFLP. The *PIK3CA* ^*H1047R*^ mutant plasmid was spiked into wild-type plasmid at different ratios, and analyzed by PCR-RFLP method (top) and direct sequencing (bottom). M: DL500 DNA marker

In order to evaluate the specificity of this method, we synthesized three sequences for detection, including two sequences with clinically present *PIK3CA* exon-20 mutation patterns (CGT and CTT) and one with a wild-type pattern (CAT). As shown in Fig. 3a, the sequence containing CAT showed two fragments (96 and 30 bp), indicating wild-type *PIK3CA*; while the sequences containing CGT or CTT showed a 126 bp fragment, even after the digestion of FspI, indicating the mutant-type *PIK3CA*.

To assess the sensitivity of the method, we constructed the plasmids carrying the 126-bp fragment of wild-type and homozygous mutant-type *PIK3CA* and diluted the homozygous mutant plasmid in increasing concentrations of the wild-type plasmid to mimic tumor heterogeneity. As shown in Fig. 3b (top), the 126-bp fragment band representing the *PIK3CA*^*H1047R*^ mutation gradually decreased with decreasing proportions of the mutant sequence, but it was still detectable, even at mutation concentrations as low as 1:256, indicating that the sensitivity of our PCR-RFLP method was approximately 0.4 %. In contrast, DNA sequencing was not able to detect the *PIK3CA*^*H1047R*^ mutation when present at approximately 25 % (1:4) of the total mixture, suggesting that its detection sensitivity was approximately 25 % (Fig. 3b, bottom).

### Detection of mutant *PIK3CA*^*H1047R*^ in clinical CRC samples

**Fig. 4:**
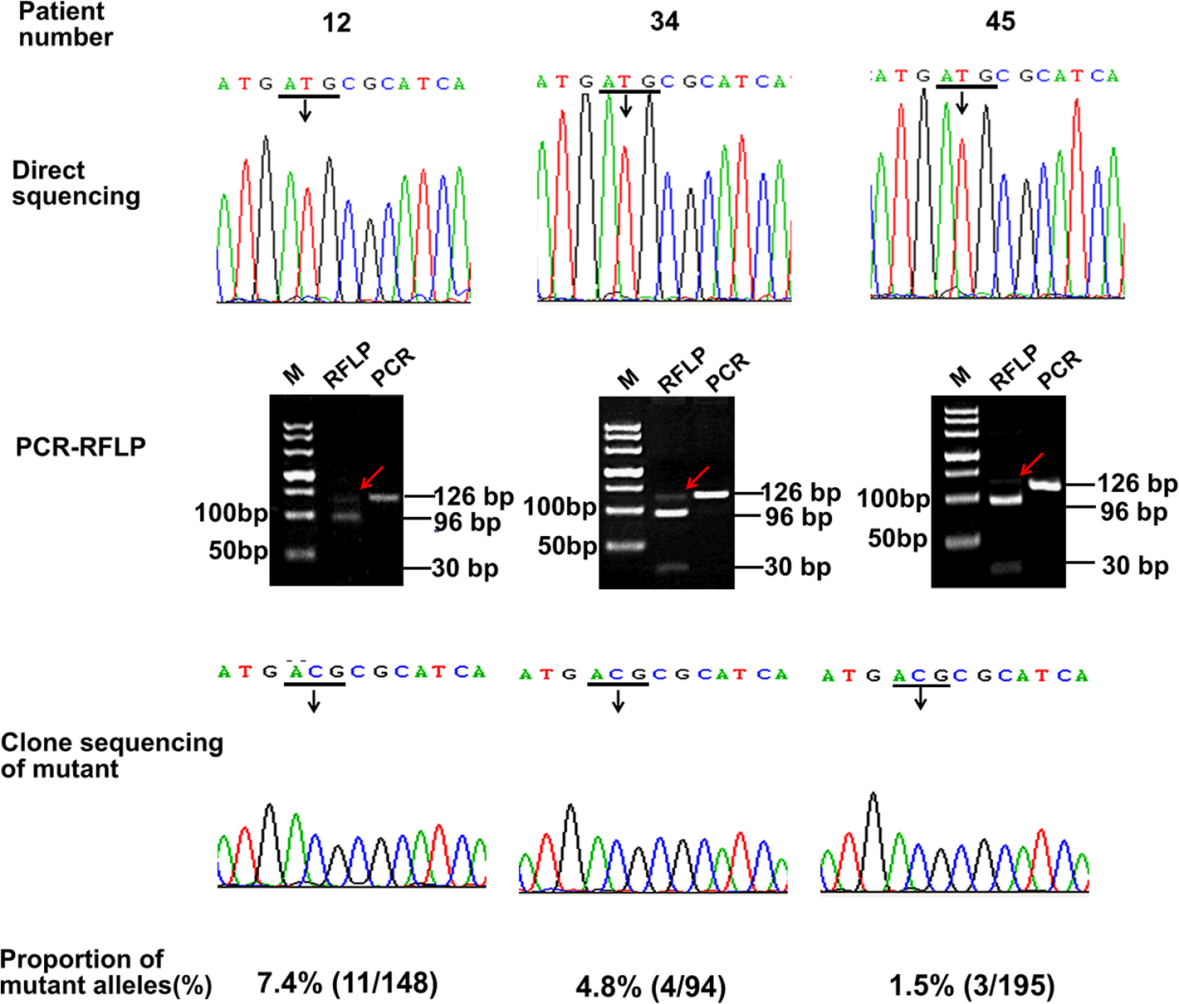
Three representative detection results of PCR-RFLP and clone sequencing for CRC FFPE samples. The mutant bands in PCR-RFLP electrophotograms are indicated by the red arrows. The mutant sits of *PIK3CA* ^*H1047R*^ in sequencing results were indicated by the black arrows. M: DL500 DNA marker

In order to investigate the clinical applicability of the PCR-RFLP method, 86 FFPE tissue sections from CRC patients were analyzed. As a result, the PCR-RFLP method identified 16.5 % *PIK3CA*^*H1047R*^ mutation, higher than the frequency identified by DNA sequencing (8, 9.3 %), among which there were six *PIK3CA*^*H1047R*^ mutant cases that failed to be detected by DNA sequencing. To determine the accuracy of our method, these six CRC cases were further analyzed by clone sequencing. Three representative results are shown in Fig. 4, and revealed the method’s capability to detect at least 1.5 % of the *PIK3CA*^*H1047R*^ mutation in CRC specimens, which was far below that of direct sequencing.

We further explored the correlation between the CRC patients’ clinicopathological data and the mutation status of the *PIK3CA*^*H1047R*^. Statistical analysis of the PCR-RFLP results revealed that the *PIK3CA*^*H1047R*^ mutation was not significantly associated with gender, tumor size, tumor location, Dukes stage and lymph node status (Table 1). However, levels of the *PIK3CA*^*H1047R*^ mutation were significantly higher in patients who were older than 60 years in comparison with patients ≤60 years of age (24.5 vs. 5.4 %, *P* = 0.018). Direct sequencing did not reveal the relationship between the *PIK3CA*^*H1047R*^ mutation and age. In addition, the *PIK3CA*^*H1047R*^ mutation was negatively associated with the degree of differentiation by both PCR-RFLP and direct sequencing method.

**Table 1:** Clinicopathological characteristics and *PIK3CA* ^*H1047R*^ mutation status in 86 CRC cases

Category	Total (n)	Direct sequencing		PCR-RFLP	
		Mutation (%)	*P*-value	Mutation (%)	*P*-value
Number of patients	86	8 (9.3)		14 (16.3)	
Age (years)			0.145		0.018*
>60	49	7 (14.3)		12 (24.5)	
≤60	37	1 (2.7)		2 (5.4)	
Gender			0.275		0.498
Male	53	3 (5.7)		7 (13.2)	
Female	33	5 (15.2)		7 (21.2)	
Tumor size			0.924		0.702
>5 cm	47	5 (10.6)		7 (14.9)	
≤5 cm	39	3 (7.7)		7 (17.9)	
Tumor location			0.428		0.564
Colon	37	5 (13.5)		7 (18.9)	
Rectum	49	3 (6.1)		7 (14.3)	
Tumor differentiation			0.001*		< 0.0001*
Well or Moderate	67	2 (3.0)		4 (6.0)	
Poor	19	6 (31.6)		10 (52.6)	
Dukes’ stage			1.000		1.000
A + B	60	6 (10)		10 (16.7)	
C + D	26	2 (7.7)		4 (15.4)	
Lymph node metastasis			0.603		0.708
+	34	2 (5.9)		4 (11.8)	
−	69	6 (8.7)		10 (14.5)	

*Statistically significant (*p* < 0.05)

^*^ statistically significant (*p *< 0.05) 

### Detection of the KRAS, BRAF and PIK3CA mutations in CRC specimens using the PCR-RFLP method

**Fig. 5:**
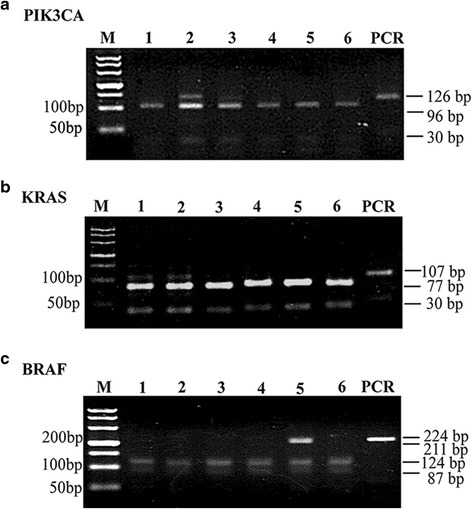
Detection of *PIK3CA*, *KRAS* and *BRAF* mutations by individual PCR-RFLP methods in CRC samples. **a**: Detection of the *PIK3CA* mutation using our PCR-RFLP. **b**: Detection of the *KRAS* mutation using PCR-RFLP. A 107-bp fragment of *KRAS* was amplified, followed by digestion with MvaI and analysis by 3 % agarose gel electrophoresis. The MvaI digestion of wild-type *KRAS* yielded two 77- and 30-bp bands, while the mutant-type remained intact (107 bp). **c**: Detection of the *BRAF* mutation using PCR-RFLP. A 224-bp fragment of *BRAF* was amplified, and followed by digestion with TspRI and analysis by 3 % agarose gel electrophoresis. The TspRI digestion of wild-type *BRAF* yielded three 124-, 87- and 13-bp bands, while the mutant-type yielded two 211- and 13-bp bands. M: DL500 DNA marker

*KRAS*, *BRAF* and *PIK3CA* are considered to have negative effects on the response to anti-EGFR MoAbs in CRC. To investigate the possibility of detecting three gene mutations by the PCR-RFLP method, six of 86 case samples were analyzed. The PCR-RFLP electrophoresis results are shown in Fig. 5. For *PIK3CA*, 96 and 30-bp fragments were detected in all specimens, while an extra band at 126-bp was clearly detected in specimens 2 and 3, suggesting they carried *PIK3CA* mutations. For *KRAS*, specimens 1 and 2 had a 107-bp fragment as well as 77- and 30-bp fragments, suggesting the mutant-type of *KRAS*. For *BRAF*, in addition to an extra band at 211-bp in specimen 5, the other specimens had 124- and 87- bp fragments, suggesting only the specimen 5 contained *BRAF* mutations. The mutation status of *KRAS*, *BRAF* and *PIK3CA* are summarized in Table 2 (WT: wild-type, M: mutant-type).

**Table 2:** Gene status in different CRC FFPE samples by PCR-RFLP method

Sample	*PIK3CA*	*KRAS*	*BRAF*
1	WT	M	WT
2	M	M	WT
3	M	WT	WT
4	WT	WT	WT
5	WT	WT	M
6	WT	WT	WT

## Discussion

CRC is one of the most common human malignant diseases and is a leading cause of cancer-related deaths worldwide. Metastases are the major cause of death in CRC patients [32]. Recently, targeted therapies against EGFR, such as cetuximab and panitumumab, have improved the survival of patients with metastatic CRC (mCRC) [33]. However, less than 20 % of unselected mCRC patients can truly benefit from the anti-EGFR MoAb treatment [34], highlighting the need to determine those who are more likely to obtain a clinical benefit from this targeted therapy. *KRAS* is the first gene proven to be a predictive biomarker for resistance to the anti-EGFR MoAb treatment, and *BRAF* has also been demonstrated to be a response predictor [35]. Recently, active *PIK3CA* mutations were found to be able to predict resistance to anti-EGFR MoAbs. There are two major mutational hotspots in exons 9 (E542K, E545K) and 20 (H1047R) of the *PIK3CA* gene, and recent studies have suggested that the *PIK3CA*^*H1047R*^ mutation had a closer relationship with anti-EGFR MoAb treatment [10]. Thus, the accurate identification of the *PIK3CA*^*H1047R*^ mutation status is very crucial for guiding personalized therapy.

To effectively detect the *PIK3CA*^*H1047R*^ mutation status, we developed a novel PCR-RFLP method by creating an FspI restriction site. The results showed that the PCR-RFLP method could distinguish the wild-type and mutant-type *PIK3CA*^*H1047R*^ with complete agreement with the results obtained by DNA sequencing in different CRC cell lines. The specificity of the method was verified by the analysis of various patterns of the *PIK3CA*^*H1047*^ mutation, and its high detection sensitivity was demonstrated using various quantities of mutation fragments spiked into wild-type fragments, achieving a detection limit as low as 0.4 %, which is significantly superior to direct sequencing (25 %). Current data in clinical trials show that not all patients grouped as wild-type for defined genes benefit from molecular targeted therapies [36]. There are several reasons for it, such as the presence of other undefined gene alterations [37, 38], but it is possible that the employed methods with the limited sensitivity may fail to detect the low-abundance mutations, resulting in incorrect classifications. Molinari et al. reported that compared with direct sequencing, 13 additional *KRAS* mutations were identified using highly sensitive methods, which all were non-responsive to anti-EGFR therapies [39]. In this study, the *PIK3CA*^*H1047R*^ mutation in 86 patients was analyzed by PCR-RFLP. As a result, we revealed the *PIK3CA*^*H1047R*^ mutation in 16.3 % of the CRC samples and this ratio is significantly higher than the result we obtained using direct sequencing (9.3 %), and the lowest mutation was 1.5 %. The results demonstrated that our method could detect low-abundance *PIK3CA*^*H1047R*^ mutations and thus offer accurate guidance for personalized treatment. In addition, the detection sensitivity is expected to increase further using PAGE electrophoresis-based silver staining instead of EB staining [20].

The results of the analysis of clinicopathological characteristics from 86 CRC tissues revealed a significant correlation between the *PIK3CA*^*H1047R*^ mutation and the patient’s age. Patients over 60 years of age tend to show significantly more *PIK3CA*^*H1047R*^ mutations than patients under 60 years of age (24.5 vs. 5.4 %, *P* = 0.018). To the best of our knowledge, this is the first report to reveal the significant association between the *PIK3CA*^*H1047R*^ mutation and the patient’s age. This may possibly be due to the high detection sensitivity of the PCR-RFLP method because there was no consistent statistically significant difference found by direct sequencing. This result further suggests that a detection method that can resolve low abundance mutations might provide a better understanding of the clinical significance of a given gene mutation. Some previous studies found that the *PIK3CA*^*H1047R*^ mutation is a late event of CRC progression [7]. Additionally, according to the tumorigenesis theory, older patients tend to accumulate more types of gene mutations [40]. Because older patients possibly encounter more *PIK3CA*^*H1047R*^ mutations and do not respond to targeted therapy, an improved prognosis might be achieved by the preferential attention of the patient subpopulation at the early stage of CRC or below 60 years in clinically targeted therapy. Of course, much more data from clinical settings is needed to verify this conclusion. In addition, statistical analysis showed that patients with poorly differentiated tumors are much more likely to have the *PIK3CA*^*H1047R*^ mutation (poor 52.6 % vs. moderate/well 6.0 %, *P* = 0.000), which is in agreement with a recent report [41]. Other clinicopathological characters, such as Dukes stage, tumor size, tumor location, gender, and lymph node status showed no relationship with the *PIK3CA*^*H1047R*^ mutation.

In CRC, *KRAS* and *BRAF* mutations have been proven to be predictors of the therapeutic efficiency of anti-EGFR therapy. In 2010, De Roock and co-workers [8] demonstrated that the *PIK3CA*^*H1047R*^ mutation might be a new potential response predictor after *KRAS* and *BRAF* for resistance to anti-EGFR mAbs. However, until now, the clinical significance of *PIK3CA* mutations in terms of the prediction of the response to anti-EGFR therapy still remains incompletely understood, partly due to the lack of highly effective approaches for detecting related gene mutations in combination [42]. To explore whether our *PIK3CA*^*H1047R*^-specific PCR-RFLP can be utilized together with the reported PCR-RFLP methods for *KRAS* and *BRAF*, the three gene mutations existing in six CRC specimens were analyzed in combination by individual PCR-RFLP. The results showed that four specimens, except for specimens 4 and 6, carried mutations in different genes, which all were confirmed by DNA sequencing (data not shown), indicating that the PCR-RFLP method is able to accurately detect multi-gene mutations in combination. Like other researchers, we also found that either *PIK3CA* or *BRAF* were present in some wild-type *KRAS* specimens, such as *PIK3CA* in specimen 3 and *BRAF* in specimen 5, suggesting that it is necessary in clinical practice to investigate the state of the other two genes in *KRAS* wild-type patients. In addition, several previous reports demonstrated that *KRAS* and *BRAF* are mutually exclusive in CRCs [43, 44]. In our study, we also did not find their co-existence, but the concomitant mutation in *KRAS* and *PIK3CA* was detected in specimen 2. Nevertheless, their effect on the response to targeted treatment still needs to be verified further. All of the mutations detected by PCR-RFLP were confirmed by direct sequencing except for *PIK3CA* in specimen 3, which was verified by clone sequencing later and displayed a lower mutant frequency of 2 %. Notably, this *PIK3CA* was the only detectable mutation in the specimen, which suggested that a combinatorial PCR-RFLP strategy with high sensitivity may provide more accurate information to understand the clinical significance of gene mutations in spite of limited specimen involvement.

## Conclusions

In summary, we developed a novel PCR-RFLP method to detect the *PIK3CA*^*H1047R*^ mutation by creating an FspI restriction endonuclease recognition site. This method is able to resolve wild-type and mutant-type *PIK3CA*^*H1047R*^ with high specificity and sensitivity, allowing the low abundance mutation of 0.4 % to be detected. Additionally, this method has several advantages over other methods, such as simple operation and suitability in a routine laboratory. Using this method, 86 cases of CRC specimens were detected with high efficiency, with an excessively positive rate of *PIK3CA*^*H1047R*^ mutations relative to that using DNA sequencing. Based on this, a positive correlation between the *PIK3CA*^*H1047R*^ mutation and the patient’s age was found, which might be helpful in guiding targeted therapy. In addition, the approach was combined with PCR-RFLP methods for *KRAS* and *BRAF* together and achieved high sensitivity and the accurate detection of multiple gene mutations in parallel for CRC tissues. Overall, this method could become a promising tool for guiding personalized tumor therapy and exploring the clinical applicability of the *PIK3CA*^*H1047R*^ mutation.

## Abbreviations

CRC, colorectal cancers; FBS, fetal bovine serum; FFPE, formalin-fixed paraffin-embedded; gDNA, genomic DNA; mCRC, metastatic CRC; PCR-RFLP, Polymerase chain reaction-restriction fragment length polymorphism; PI3Ks, phosphatidylinositol 3-kinases
